# Integrative and Mechanistic Approach to the Hair Growth Cycle and Hair Loss

**DOI:** 10.3390/jcm12030893

**Published:** 2023-01-23

**Authors:** Nicole Natarelli, Nimrit Gahoonia, Raja K. Sivamani

**Affiliations:** 1 Morsani College of Medicine, University of South Florida, Tampa, FL 33602, USA; 2 College of Osteopathic Medicine, Touro University, 1310 Club Dr, Vallejo, CA 94592, USA; 3 College of Medicine, California Northstate University, 9700 W Taron Dr, Elk Grove, CA 95757, USA; 4 Integrative Skin Science and Research, 1495 River Park Drive, Sacramento, CA 95819, USA; 5 Pacific Skin Institute, 1495 River Park Dr Suite 200, Sacramento, CA 95815, USA; 6 Department of Dermatology, University of California-Davis, 3301 C St #1400, Sacramento, CA 95816, USA

**Keywords:** hair loss, androgenic, alopecia, hair thinning, anagen, telogen, hair cycle

## Abstract

The hair cycle is composed of four primary phases: anagen, catagen, telogen, and exogen. Anagen is a highly mitotic phase characterized by the production of a hair shaft from the hair follicle, whereas catagen and telogen describe regression and the resting phase of the follicle, respectively, ultimately resulting in hair shedding. While 9% of hair follicles reside in telogen at any time, a variety of factors promote anagen to telogen transition, including inflammation, hormones, stress, nutritional deficiency, poor sleep quality, and cellular division inhibiting medication. Conversely, increased blood flow, direct stimulation of the hair follicle, and growth factors promote telogen to anagen transition and subsequent hair growth. This review seeks to comprehensively describe the hair cycle, anagen and telogen balance, factors that promote anagen to telogen transition and vice versa, and the clinical utility of a variety of lab testing and evaluations. Ultimately, a variety of factors impact the hair cycle, necessitating a holistic approach to hair loss.

## 1. Overview of Hair Cycle

Hair follicles differ in size and shape depending on location, although they are characterized by the same structural components [1]. The hair shaft is produced by proliferating matrix cells found in the hair bulb, with melanocytes interspersed and responsible for pigmentation. Differentiation and upward movement contribute to the growing hair shaft, whose cortex is composed of intermediate filaments and proteins [1]. Located at the follicle base, the dermal papilla controls the number of matrix cells and subsequently the size of hair.

Hair growth occurs in a continuous process characterized by four phases: anagen, growth; catagen, regression; telogen, rest; and exogen, shedding. Individual hair follicles cycle independently, with each hair follicle undergoing ten to thirty cycles in a lifetime [2]. While most individuals have about 100,000 scalp hairs at any time, normal shedding occurs at a rate of 100 to 150 telogen hairs per day [2]. As some hairs reside in the anagen phase while others are resting or shedding, the density and total hair strand number remains relatively stable in healthy conditions.

As the longest phase of the hair cycle, anagen lasts about two to eight years among scalp hair, although various factors can promote anagen to telogen transition, reducing growth while fostering rest and eventual shedding [2]. Anagen is characterized by the production of an entire hair shaft from hair follicles; as such, hair length in the absence of cutting directly corresponds to anagen length. For example, whereas scalp hair follicles reside in anagen for two to eight years, eyebrow hair follicles reside in anagen for only two to three months [1]. However, anagen phase length decreases with age, resulting in weaker and thinner hair over time [3]. Similarly, the proportion of follicles in the anagen phase declines with age [4]. Importantly, anagen hair shedding resulting from premature termination of anagen growth or anagen arrest secondary to insult is never normal.

The catagen phase represents the transition from anagen to telogen, lasting about two weeks. Throughout the catagen phase, hair follicles regress and detach from the dermal papilla, the population of mesenchymal cells in hair follicles, resulting in epithelial cell apoptosis in the bulb of the follicle [3,5]. Following catagen, the dermal papilla moves upward towards the hair-follicle bulge. If the dermal papilla is unable to reach the bulge during catagen, follicle cycling terminates resulting in loss of hair [1]. The telogen resting phase follows, lasting about two to three months. At any time, about 9% of total scalp hair resides in the telogen phase [4], in comparison to 40–50% of total hair on the trunk [1]. While old hair is resting, new hair begins to develop at the base of the hair follicle, eventually pushing old hair out. However, if anagen enters the resting phase prematurely, excessive shedding and thinning can occur, known as telogen effluvium (TE). Conversely, reducing the percent of hair follicles residing in the telogen phase manages hair loss [1]. Lastly, exogen describes the termination of telogen and the initiation of anagen. During this period, newly developing hair continues to grow upward, pushing the old hair out, resulting in its ultimate shedding.

**Figure 1:**
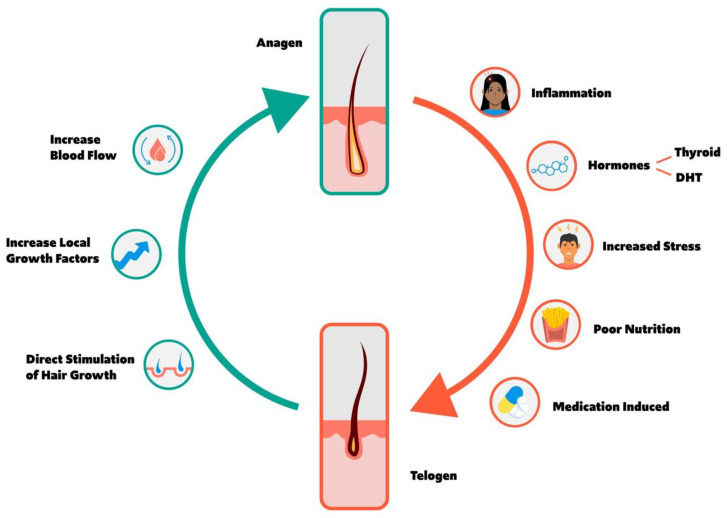
Schematic of the hair growth cycle and the factors that may influence a transition from anagen to telogen vs. telogen to anagen phase.

Figure 1 shows a hair growth cycle depiction of the balance between anagen and telogen along with the factors that may influence the hair growth cycle.

### 1.1. Anagen and Telogen Balance

The anagen to telogen ratio among healthy subjects is approximately 14:1 to 12:1 among healthy subjects [6,7]. However, various subtypes of alopecia are characteristic of decreased anagen to telogen ratios and subsequent hair shedding above the normal rate of 100–150 strands daily.

The causes of alopecia can be categorized as scarring, such as cicatricial alopecia, and non-scarring, including alopecia areata (AA), androgenetic alopecia (AnA), and TE. Alopecia areata is characterized by circular bald patches that may or may not overlap, whereas AnA refers to male- and female-pattern hair loss, characterized by the progressive shortening of anagen cycles [1]. A descriptive study of alopecia patterns among 1232 patients presenting to a clinic over the course of 25 months found diffuse alopecia to be the most prevalent form of hair loss (71.35%). A total of 14.3% of patients presented with AnA, in comparison to 11.8% with AA [8].

AA is characterized by an anagen to telogen ratio of approximately 6:4 or 5:5, and in some cases the proportion of hair follicles in telogen can exceed that of anagen [9]. The anagen to telogen ratio decreases to approximately 5:1 in AnA [6] and 8:1 in TE [7]. Thus, alopecia is fundamentally characterized by an imbalance of anagen and telogen. Importantly, a variety of factors can increase anagen to telogen transition, fostering hair loss. Conversely, factors and treatments can increase telogen to anagen transition, prompting hair growth.

## 2. Factors Increasing Anagen to Telogen Transition and Hair Loss

As a variety of factors increase the transition from anagen to telogen, it is essential to consider all possible contributing factors when presented with a general complaint of non-scarring alopecia. Obtaining a thorough history is crucial to consider the root causes of alopecia and optimize therapeutic approaches for individual cases.

### 2.1. Inflammation

Inflammation fosters anagen to telogen transition and has been associated with the progression of alopecia [10]. Inflammation has been suggested to mediate a variety of hair loss subtypes, including stress-induced hair loss, alopecia areata (AA), and male- and female-pattern hair loss, also known as androgenic alopecia (AnA). Each of these alopecia subtypes are associated with a decreased anagen to telogen ratio, as described in Section 1.1. In addition, chronic, systemic inflammatory disorders can cause TE, characterized by premature progression from anagen to telogen [7].

A 1975 study observed lymphocytes and histiocytes, markers of chronic inflammation, in approximately half of 347 tissue specimens collected from patients with male-pattern androgenetic alopecia (MPAnA) [11]. Furthermore, significant perivascular infiltration of mast cells was observed in 40% of specimens. Similarly, a study found moderate to severe inflammation with lymphocytic and histiocytic infiltrates in 36% of 106 biopsy specimens from patients with MPAnA, compared to 9.1% of control specimens [12]. In a separate study, the same author observed 36.8% of specimens from 412 MPAnA and female-pattern androgenetic alopecia (FPAnA) patients depicting moderate or severe perifollicular inflammation, compared to 9.1% of control specimens [13]. Similarly, in 2011 authors conducted scalp biopsies with 52 FPAnA patients and observed lymphocytic folliculitis targeting bulge epithelium in many cases [14]. These studies describe the association between chronic inflammatory cell infiltrate and AnA, suggesting inflammation may partially mediate pathophysiology and contribute to increased anagen to telogen transition. Furthermore, lotion application consisting of antimicrobial and antifungal agents were found to decrease the number of activated T cells over the course of treatment among patients with MPAnA, highlighting inflammation as a therapeutic target [15].

In addition, a mouse study found that inflammatory events in the hair follicle environment may mediate stress-induced hair loss. Authors observed perifollicular macrophage cluster and excessive mast cell activation in the hair follicle environment of stressed mice, suggesting inflammatory and immunological events of the stressed mouse may contribute to stress-induced hair loss [16]. In addition, the authors found that stress-related hair growth inhibition can be replicated by substance P, which exhibits proinflammatory effects in immune and epithelial cells [17] in non-stressed mice [16]. Similarly, another murine study found an increased number of substance P-immunoreactive nerve fibers in the skin during the early stages of AA, and substance P cutaneous application led to a significant increase in mast cell degranulation and accelerated catagen [18]. These studies suggest that inflammation may mediate both stress-induced hair loss and AA, with proinflammatory substance P as an important regulator.

In addition to murine studies, human studies have described inflammation associated with AA, which is associated with a decreased anagen to telogen ratio of 6:4 or 5:5 [9], compared to the normal ratio of 12:1. A 2012 study described greater serum immunoglobulin (Ig)E levels among patients with diffuse or patchy AA [19]. Compared to patchy AA biopsies, diffuse biopsies depicted more intense mononuclear, eosinophil, CD3+, and CD8+ T cell infiltration around hair bulbs, and IgE levels positively correlated with infiltration intensity [19]. A 2013 study observed dermal inflammatory infiltration and epithelial cell damage of the hair follicle infundibulum in early AA lesions [20]. A total of 40% of patients showed eosinophilic infiltration, which was positively correlated with elevated serum IgE levels, severe perivascular lymphocytic inflammation in the upper dermis, and peri-follicular infiltration [20].

These studies effectively associate inflammatory infiltrate and fibrosis with alopecia conditions characterized with a decreased anagen to telogen ratio. Inflammation is an important factor mediating anagen to telogen transition.

### 2.2. Hormones

A variety of hormones have been shown to impact the hair cycle and mediate hair growth, including thyroid hormones, dihydrotestosterone (DHT), estrogen and testosterone.

#### 2.2.1. Thyroid Hormone

Hypo- and hyperthyroidism can cause reversible, diffuse hair loss [2] and can promote premature transition from anagen to telogen, potentially resulting in telogen effluvium. In fact, diffuse hair loss may be the only presenting sign of thyroid dysfunction [8]. A study published in 2013 assessed alopecia patterns related to thyroid dysfunction among all patients presenting to a clinic from December 2007 to December 2009 [8]. Thyroid dysfunction, based on a thyroid-stimulating hormone reference range, was observed most frequently in AA and diffuse alopecia patients among those aged 0–20 and 21–40 years, and in AA and AnA patients among those 40 years and older. A greater association between thyroid dysfunction and alopecia was observed with increasing age [8].

The mechanisms of aberrant thyroid hormone levels and hair loss have been described. The deletion of murine thyroid hormone nuclear receptors has been shown to impair epidermal proliferation and hair growth [21]. In addition, a 2015 study found that mice with deficient thyroid hormone receptors had increased label-retaining cells in the bulges, the hair follicle stem cell niche, resulting in reduced activation of stem cells and accumulation in bulges [21]. Authors concluded that thyroid hormone signaling is necessary for proper mobilization of stem cells from the hair bulge, and improper stem cell signaling may mediate hair loss associated with thyroid hormone deficiencies. In addition, prolonged thyroid hormone stimulation has been shown to promote progenitor cell differentiation and subsequent stem cell depletion [21]. As such, both deficient and excessive levels of thyroid hormones can contribute to anagen to telogen transition and hair loss. Thyroid-stimulating hormones (TSH) and thyroxine levels should be obtained as part of a standard work-up for non-scarring alopecia.

#### 2.2.2. Dihydrotestosterone

Dihydrotestosterone (DHT) is an androgenic steroid hormone produced via the action of 5-alpha-reductase type 2, which converts testosterone to DHT at target tissues. While androgens increase hair follicle size in androgen-dependent locations, they can result in miniaturization of scalp follicles later in life and contribute to AnA [1]. DHT is a pure androgen, as it cannot be converted to estrogen [22]. In addition to the sexual development of males, DHT promotes male-pattern hair loss and is implicated in MPAnA pathophysiology. Upon binding to androgen receptors in the hair follicle, DHT promotes the shortening of the anagen phase and elongation of the telogen phase [23], resulting in enhanced apoptosis of hair cells and thus hair loss [24]. A mouse-model study found that DHT promoted premature hair regression, hair miniaturization, loss of hair density, and altered hair morphology in male mice, with partial reversal with an androgen receptor antagonist, bicalutamide [25].

Unsurprisingly, men with MPAnA may be genetically predisposed to greater levels of 5-alpha-reductase and hair follicle androgen receptor activity [25]. In addition, those with 5-alpha-reductase enzyme deficiencies are less likely to develop MPAnA. The role of DHT in the promotion of transition to telogen and MPAnA pathophysiology justifies the use of oral 5-alpha-reductase inhibitors, such as finasteride, in the management of hair loss. Two one-year trials encompassing 1553 men with male-pattern hair loss found 99% of subjects to show decreased progression or the reversal of hair loss with oral finasteride. In addition, authors observed clinically significant increases in hair count with oral finasteride treatment compared to placebo (*p* < 0.001) [26]. However, as DHT is an androgen, treatment with 5-alpha-reductase inhibitors and a reduction in DHT levels has the rare side effects of sexual dysfunction and diminished libido [22].

Interestingly, however, the usefulness of collecting serum DHT levels in a routine hair loss work-up has been debated. A 2014 study analyzed serum DHT concentrations among 19 women and 9 men with AnA, in addition to 17 healthy women and 4 healthy men without hair loss [27]. Although increased serum DHT concentrations were observed in patients with AnA, as expected, increased serum DHT concentrations were also observed in the control group with no statistically significant difference between groups [27]. In addition, the authors found no correlation between DHT concentrations and the progression of alopecia, although the study is limited by a small sample size. The authors concluded that rather than serum DHT concentration, the genetically determined sensitivity of hair follicles to DHT may mediate DHT-associated hair loss [26]. However, these results are conflicting with another study including 178 patients with MPAnA and 61 healthy controls, which found a significantly greater level of DHT in MPAnA patients than normal controls. Yet, similarly to the prior study, authors found no significant difference in serum androgen levels based on hair loss severity [28].

In conclusion, there is questionable utility for routine serum DHT testing for patients with hair loss. However, it is necessary to understand the role of DHT in the pathophysiology of hair loss, as the enzyme converting testosterone to DHT, 5-alpha-reductase, is an effective therapeutic target for MPAnA.

#### 2.2.3. Estrogen to Testosterone Ratio

Numerous studies have assessed the effects of testosterone and estrogen in isolation on hair parameters, including anagen phase length. While testosterone conversion to DHT can promote hair loss, estrogen has been postulated to have protective effects against hair loss based on differential observations of hair parameters throughout pregnancy, postpartum, and menopause, each characterized by estrogen concentration differences. In pregnancy, characterized by high levels of estrogen, hair growth, and hair diameter increases while the hair shedding decreases [29]. These observations have been attributed to estrogen, although other pregnancy-related changes, such as increases in human chorionic gonadotropin, progesterone, prolactin, growth factors, and cytokines, may additionally contribute [29]. In contrast, a decrease in estrogen and progesterone following delivery is associated with postpartum TE. Furthermore, estrogen depletion characteristic of menopause is associated with FPAnA, with decreased hair density and diameter, and decreased anagen phase length [29]. The protective role of estrogen in hair loss is further supported by the observation that the frontal hairline of women, often spared in FPAnA, depicts a relatively increased level of aromatase, the enzyme responsible for the conversion of androgens to estrogen [30].

However, rather than serum values in isolation, research has suggested that a decreased estrogen to testosterone ratio, rather than absolute values of either hormone, may instead contribute to FPAnA [31]. Serum levels of luteinizing hormone (LH), follicle-stimulating hormone (FSH), estradiol, free and total testosterone, sex hormone binding globulin (SHBG) and dehydroepiandrosterone sulfate (DHEAS) were studied among 20 premenopausal women with FPAnA and 9 healthy controls [31]. Absolute values of androgens were normal in both groups, although the patients with FPAnA depicted reduced estradiol to free testosterone and estradiol to DHEAS ratios. The authors therefore concluded that the estradiol to free testosterone ratio may contribute to FPAnA.

Thus, rather than increasing the absolute values of estrogen, deliberately increasing the estrogen to testosterone ratio may be an effective therapeutic strategy. Estrogen replacement therapy has been assessed for the management of alopecia, both in female and male patients. A case report depicted extensive hair regrowth in a male-to-female transition candidate with AnA treated with estradiol supplementation and estrone solution, although simultaneous treatment with minoxidil inhibits the ability to make direct conclusions of the efficacy of estrogen replacement. However, the patient was also treated with anti-androgenic Aldactone (spironolactone), so it is possible extensive hair regrowth occurred due to increased estrogen to testosterone activity [32].

In addition, a study compared the efficacy of two oral contraceptives, one containing antiandrogenic chlormadinone acetate with synthetic estrogen and another containing synthetic progestin with synthetic estrogen, on acne parameters; the authors also reported resolution rates of alopecia [33]. Alopecia resolution rates were 86% and 91% among those receiving chlormadinone/synthetic estrogen and synthetic progestin/synthetic estrogen, respectively. This suggests anti-androgenic activity coupled with estrogen replacement, and thus an increased estrogen to testosterone activity ratio may be as effective as an estrogen and progestin replacement for the resolution of alopecia.

Ultimately, larger, controlled studies are necessary to assess the efficacy of therapy specifically targeting the estrogen to testosterone ratio. However, prior reports suggest the estrogen to testosterone ratio may be of more value than the absolute values of either hormone, which may further explain why sex hormone concentrations often fail to correlate with reported alopecia symptoms [34].

### 2.3. Stress

The association of stress and hair loss has been widely documented. As previously described, Arck et al. suggested that substance P-dependent inflammatory pathways may mediate stress-induced hair loss [16]. In 1998, a case-control study used the Social Readjustment Rating Scale to compare stress among twenty-five women who experienced recent hair loss compared with twenty-five healthy controls [35]. Compared to ten control subjects, twenty-two of those experiencing unexplained hair loss reported high stress, resulting in an odds ratio of eleven; based on this study, authors concluded that women experiencing high stress are eleven times more likely to experience hair loss [35]. However, this study is limited by its small sample size and potential recall bias. Still, this study depicts an early documentation of the association of stress and hair loss.

Stress can foster anagen to telogen transition and is closely related to telogen effluvium, with resulting telogen elongation [36]. Furthermore, cortisol, the primary stress hormone, has been shown to affect cyclic regulation of the hair cycle and proteoglycan synthesis [23]. The effects of cortisol on the hair cycle and proteoglycans are important to understand, as elevated cortisol levels have been observed in both men and women with androgenetic alopecia in comparison to healthy controls [37,38].

Research has highlighted the importance of proteoglycans, such as versican and decorin, and glycosaminoglycans in normal hair follicle and hair cycle functioning. For example, while versican functions to protect cells from oxidative stress-induced apoptosis, decorin acts as an anagen inducer, promoting hair growth [23]. However, high cortisol levels have been shown to exhibit damaging effects on proteoglycans in the hair follicle, with reduced synthesis and increased breakdown [39]. Thus, cortisol inhibition may promote anagen and hair growth via increased proteoglycan concentrations.

A study compared shampoo containing 2% ketoconazole, an antifungal cortisol inhibitor, with unmedicated shampoo among 39 patients with MPAnA. Medicated shampoo increased hair density and the size and proportion of hair follicles residing in the anagen phase, both in isolation and in combination with minoxidil [40]. Similarly, a 2007 study including six patients with MPAnA found hair regrowth with 2% ketoconazole topical lotion [41]. Interestingly, one patient stopped using the lotion and depicted hair loss recurrence three months later, suggesting continual ketoconazole application is required for maintenance of hair regrowth. In addition, the authors found that ketoconazole may promote hair regrowth via both androgen-dependent and androgen-independent mechanisms [41].

Lastly, a 2019 study compared the efficacy of 2% topical ketoconazole in comparison to 2% minoxidil among patients with FPAnA [42]. Whereas a significant difference between baseline and months 4 and 6 was observed among those receiving topical minoxidil, significant improvement with ketoconazole was observed only at month 6, suggesting delayed treatment efficacy with ketoconazole. However, whereas treatment-related side effects were reported among 55% of those receiving minoxidil, side effects were reported in only 10% receiving ketoconazole, and there was no difference in patient satisfaction between the groups [42]. These studies highlight the potential therapeutic role of cortisol inhibition on hair regrowth in patients with both male and female-pattern androgenetic alopecia, although additional large, randomized controlled trials are needed to better assess efficacy.

Work has highlighted the role of stress and cortisol in hair cycle dysregulation and hair loss. Unfortunately, stress can often act as an initiating event and a resulting outcome of hair loss, further perpetuating hair loss. However, cortisol-inhibition may be an effective therapeutic target for the treatment of androgenetic alopecia.

### 2.4. Nutritional Deficiencies

Proper nutrition is essential for anagen and telogen balance, and caloric or nutritional deficiency can negatively impact hair structure, growth, and pigmentation [43]. Furthermore, TE can occur following rapid weight loss or reduced protein intake, and diffuse alopecia may be a presenting sign of nutritional deficiency [44]. Studies have found associations between nutritional deficiency and a variety of types of hair loss, including chronic TE, AnA, and AA [44]. A variety of nutritional components have been evaluated for their effect on hair structure and growth, including a variety of vitamins and minerals, in addition to fatty acids and protein. There remains continued uncertainty regarding the value of nutritional supplementation for hair loss, especially among non-deficient individuals, and the over-supplementation of some nutrients may increase toxicity and even contribute to hair loss.

#### 2.4.1. Amino Acids and Protein

Protein is an important dietary source of many important vitamins, including B and E vitamins. Protein-energy malnutrition, observed in children with kwashiorkor, marasmus, and marasmic-kwashiorkor conditions, is associated with skin and hair alterations [43]. A 2017 cross-sectional study sought to determine the prevalence of a variety of nutritional deficiencies, including essential and non-essential amino acids, among one-hundred patients with TE, MPAnA, or FPAnA [45]. Deficiency of essential amino acids histidine, leucine, and valine were common among alopecia subtypes. Specifically, more than 90% of participants with AnA and 77.8% with TE exhibited histidine deficiency, and 98.2% of patients with TE exhibited leucine deficiency, in addition to all patients with FPAnA. Among non-essential amino acids, alanine and cysteine deficiencies were the most common. A total of 91.67% of patients with FPAnA, 91.18% of patients with MPAnA, and 90.74% of patients with TE had alanine deficiencies; 55.58% and 50% of patients with MPAnA and TE, respectively, exhibited cysteine deficiency [45]. The results of this study exemplify the association between select amino acid deficiency and various alopecia subtypes.

Many studies assessing amino acid supplementation for hair loss are limited based on non-disclosure of complete supplement composition and the inclusion of other nutritional components, limiting the ability to assess the direct effect of amino acid supplementation [44]. However, a 2007 study observed statistically significant improvement and normalization of mean anagen hair rate following a six-month treatment with an oral supplement composed of L-cystine, medicinal yeast, and pantothenic acid (vitamin B5) [46].

Studies have additionally assessed the role of marine proteins on hair loss [47,48]. A 2015 study randomized female participants with self-reported hair thinning to receive either an oral supplement containing marine proteins and glycosaminoglycans (N = 30) or a placebo supplementation (N = 30) [47]. Twice daily treatment supplementation for 90 days resulted in a significant increase in terminal hair number compared to baseline and placebo (*p* < 0.0001). In addition, significantly less hair shedding, greater Self-Assessment, and Quality of Life Questionnaire scores were observed with oral protein supplementation. Similarly, a 2015 study observed significantly decreased hair shedding and significantly increased hair diameter following supplementation with a marine protein-based dietary supplement compared to placebo [48].

Collectively, these studies demonstrate relative amino acid deficiencies among patients with various alopecia subtypes. Furthermore, oral protein-based supplementation depicts promising results highlighting the importance of a nutritional approach to the management of hair loss among some patients. It remains unclear whether protein or amino-acid supplementation is necessary for all alopecia patients, with or without marked deficiencies. Another unclear question is whether supplementation with select amino acids may provide benefit over a general protein supplement.

#### 2.4.2. Fatty Acids

In addition, studies have suggested that omega-3 and omega-6 fatty acid deficiencies may contribute to an increased proportion of hair follicles residing in the telogen phase and resulting alopecia. Arachidonic acid, an omega-6 fatty acid, has been shown to promote growth factor expression implicated in hair growth, such as fibroblast growth factor (FGF)-7 and FGF-10, in murine models [49]. In addition, arachidonic acid supplementation prolonged the anagen phase and promoted hair shaft elongation. Furthermore, unsaturated fatty acids may function to inhibit 5-alpha-reductase and modify androgen activity similarly to finasteride [44].

Fatty acid supplementation reduced alopecia in self-grooming rhesus macaques [50]. In addition, topical linoleic acid application was shown to reverse scalp dermatitis, alopecia, and depigmentation of hair in one case report [51]. Lastly, a randomized controlled trial including one hundred-twenty healthy females observed a significantly reduced telogen hair percentage following 6-months of supplementation with omega 3 and 6 fatty acids, in addition to antioxidants [52]. Compared to the control group, those receiving the supplement exhibited greater promotion of anagen hair, suggesting that fatty acid supplementation may function to increase the anagen to telogen ratio.

#### 2.4.3. Vitamins

Micronutrients, including vitamins, impact the normal hair follicle cycle and foster cellular turnover of matrix cells in hair follicle bulbs [43]. Furthermore, murine models have demonstrated an increased proportion of hair follicles in anagen with dietary vitamin A supplementation [53]. However, excess vitamin supplementation has also been shown to have negative effects on hair parameters [43].

##### Vitamin A

A 2015 murine study found that vitamin A increases nuclear localized beta-catenin and WNT7A levels within the hair follicle bulge in a dose-dependent manner [53]. This suggests that the effects of dietary vitamin A on anagen induction and stem cell activation occur via increased WNT signaling. Other studies suggest retinoic acid, a metabolite of vitamin A, may regulate hair follicle stem cells in a U-shaped dose-dependent manner [54].

Interestingly, vitamin A deficiencies have not been directly associated with hair loss, although over-supplementation has [43]. A 1979 case report detailed a woman experiencing sudden hair loss, and a clinical work-up revealed excess serum vitamin A levels secondary to consumption of a daily vitamin A supplement [55]. Similarly, authors reported decreased hair count, density, and percent of anagen hairs among thirty acne vulgaris patients receiving isotretinoin, an oral derivative of vitamin A [56]. Current research suggests that although vitamin A can stimulate stem cells and induce anagen, over-supplementation and excess serum levels can have deleterious effects on hair parameters.

##### B Vitamins

B vitamins, including niacin (vitamin B3), biotin (vitamin B7), and folic acid (vitamin B9) have been implicated in hair loss. For example, in addition to the well-documented pellagra characteristic of niacin deficiency, alopecia is an additional common clinical finding associated with deficient niacin [43]. However, no studies have directly assessed niacin levels among patients presenting solely with hair loss, and studies have found no significant difference in folate levels between alopecia patients and control subjects [57,58].

Biotin, a cofactor for carboxylation enzymes with dietary sources including protein, has been more extensively assessed for effects on hair parameters, and it is included in a variety of supplements or serums intended for hair health [44]. Genetic biotin deficiency is associated with severe dermatitis and alopecia (infantile) and sparse or absent scalp, eyebrows, and eyelash hair (infantile). Similarly, acquired biotin deficiency is characterized by alopecia and brittle nails [43]. A 2016 study assessed serum biotin levels in women with self-reported hair loss and found 38% of patients reported a biotin deficiency [59]. However, this study did not include matched controls.

Despite many misconceptions, biotin functions to increase hair strength, rather than hair growth. Furthermore, biotin can interfere with troponin and thyroid testing. For example, excess serum biotin can result in a falsely low TSH level [60], and unnecessary supplementation can lead to missed cardiac events [61]. Yet, biotin supplement use has depicted increasing trends from 1999 to 2016; a cross-sectional survey study found self-reported use of biotin at 1 mg/d or greater to increase from 0.1% (95% CI 0.0–0.05%) in 1999 to 2.8% (95% CI 1.9–3.9%) in 2015–2016 [61].

Despite biotin’s popular inclusion in marketed hair supplements, there is no indication that biotin supplementation should be used among healthy individuals [43]. While biotin supplementation has shown benefit specifically among cases in which acquired or inherited causes of biotin deficiency are identified, there is insufficient evidence supporting supplementation among healthy individuals who are not deficient [62]. Thus, vitamin B testing may only be clinically useful in cases of suspected biotin deficiency, where biotin supplementation may improve the clinical condition.

##### Vitamin D

Vitamin D is an essential nutrient in the body serving many different functions. The fat-soluble vitamin can be taken orally, present in foods and dietary supplements, or can be synthesized endogenously by the body through a photochemical reaction upon sun exposure through the skin [62]. Once activated, vitamin D is able to promote the absorption of calcium in the gut, balances bone mineralization via regulation of calcium and phosphate levels, and provides other immunomodulatory functions [63]. Although the role of vitamin D in hair loss is not completely understood, one predominant theory suggests that the expression of the vitamin D receptor (VDR) is required for a normal hair cycle [64], including anagen initiation [65]. VDR was found to be expressed in epidermal keratinocytes and mesodermal dermal papilla cells, both of which make up a hair follicle [64]. Studies performed in VDR-null mice found that the dermal papilla separates from the hair follicle during the catagen phase and this causes a failure to reinitiate the anagen phase [64]. This finding has inspired many clinical trials to investigate the role of vitamin D in those experiencing hair loss.

A case-control study performed in 2021 investigated the role of vitamin D serum levels in 30 males with androgenetic alopecia [66]. Results showed significantly lower vitamin D levels in males with androgenetic alopecia compared to healthy controls (*p* < 0.01). A prospective case-controlled study conducted in 2013 also assessed vitamin D serum levels, but specifically in women with FE or FPHL [67]. Vitamin D levels were also significantly reduced in women with FE or FPHL compared to controls (*p* < 0.001) and were found to be increasingly abnormal as disease severity increased.

A review performed in 2017 summarized trials which assessed vitamin D levels in FPHL and TE [64]. Two studies that were conducted solely in patients with FPHL both revealed significantly lower vitamin D levels compared to controls. Three studies assessed vitamin D levels in patients with TE. Two of the three studies described significantly lower vitamin D levels in patients with TE, however one study conducted by Karadag et al. revealed the opposite [68]. Serum vitamin D levels were significantly higher in those with TE compared to controls (*p* < 0.01). The authors explained that this increase was likely compensatory instead of causatory to the hair loss.

Overall, low vitamin D levels in patients experiencing hair loss has been consistently reported by many studies in both men and women. Data is scarce regarding the efficacy of vitamin D supplementation or topical solution for patients with vitamin D deficiency and concomitant alopecia. However, animal model studies have found benefit with vitamin D supplementation [69], and oral vitamin D supplementation with minoxidil was found to be significantly more effective than minoxidil alone in the treatment of FPAnA [70]. Yet, oral vitamin D monotherapy did not result in significant improvement. Overall, additional studies are required to determine vitamin D supplementation efficacy, both among hair loss patients depicting deficiency and those who do not. Assessing the serum vitamin D level in patients presenting with hair loss may be beneficial but the evidence for vitamin D supplementation is supportive of use with minoxidil.

##### Vitamin E

Vitamin E is postulated to increase hair count due to its antioxidant activity and lipid peroxidation inhibition, although data is similarly lacking regarding the benefits of supplementation [71]. However, a 2010 study describes a significantly increased number of hairs among 21 subjects receiving tocotrienol supplementation compared to 17 subjects receiving a placebo supplementation; a 34.5% increase in hair number compared to baseline was observed after 8 months of tocotrienol supplementation [72].

Yet, excess vitamin E increases the risk of bleeding and decreases thyroid hormone production, which may ironically promote hair loss. For example, supplementation was associated with adverse effects on hair parameters among volunteers ingesting about 30x the recommended daily intake [44]. Interestingly, the volunteers exhibited reduced thyroid hormone levels. Additional research is necessary to determine the utility of vitamin E supplementation and effective doses that function to improve hair parameters while avoiding excess.

#### 2.4.4. Minerals

Iron, zinc, and selenium are minerals that have been implicated in hair cycle regulation. Iron deficiency is the most prevalent nutritional deficiency globally, and iron deficiency contributes to TE development [43]. Iron’s potential impact on the hair cycle is derived from its function as a cofactor for the rate-limiting enzyme of DNA synthesis [44]. Interestingly, some studies have observed low serum ferritin, the storage form of iron, among patients with chronic TE, AnA, and AA; however, other studies have found no association [44]. Section 4.1 discusses serum ferritin levels among patients with alopecia in greater detail, further describing testing utility.

Zinc, an important component of various metalloenzymes that regulate protein synthesis and cell division, has been associated with TE and brittle hair among deficient patients [44]. A study assessing serum zinc levels among 312 alopecia patients (AA, MPAnA, FPAnA, or TE) and 30 healthy controls found a significantly lower serum zinc value among patients exhibiting all types of studied hair loss compared to healthy controls (*p* = 0.002) [73].

Importantly, zinc-associated alopecia is reversible, increasing the utility of assessing serum zinc levels among patients with unexplained alopecia [44]; prior work has established the benefit of oral zinc supplementation among zinc-deficient patients with TE [74] and AA [75]. However, there is no current evidence of the efficacy of zinc supplementation for individuals experiencing hair loss who are not deficient.

Selenium, a mineral that functions in oxidative damage protection and hair follicle morphogenesis, has been associated with sparse hair growth and hair loss among deficient rats [76] and mice [77]. In humans, selenium supplementation among deficient patients led to hair re-pigmentation [78] and improvement of alopecia [79]. However, similar to other minerals, there is no evidence of the utility of selenium supplementation among non-deficient patients. Furthermore, selenium toxicity can perpetuate generalized hair loss, in addition to other symptoms such as blistering skin lesions, gastrointestinal symptoms, and memory problems [44].

### 2.5. Poor Sleep

Poor sleep has been associated with increased risk and severity of alopecia subtypes, including AA and AnA. Conversely, those with alopecia have been found to exhibit reduced sleep quality compared to controls. A 2022 study analyzed the prevalence of sleep abnormalities between 223 patients with MPAnA and 223 control subjects [80]. The authors found a significant association between severe MPAnA and three sleep profiles: total sleep time less than or equal to six hours (odds ratio (OR) = 2.16, 95% confidence interval (CI) = 1.02–4.57, *p* = 0.044); a Pittsburgh Sleep Quality Index (PSQI) score greater than 5 (OR = 3.72, 95% CI= 1.42–9.72, *p* = 0.008); and STOP-Bang score greater than or equal to 5 (OR = 3.01, 95% CI = 1.11–8.13, *p* = 0.030). The STOP-Bang score specifically assesses signs of obstructive sleep apnea, and higher STOP-Bang and PSQI scores are negative findings, suggesting an association between sleep disturbances and MPAnA [80]. Similarly, poor sleep habits is associated with increased severity of AnA [81].

A similar study assessed the prevalence of sleep disturbances among 51 AA patients and 51 age- and sex-matched controls [82]. As observed among individuals with MPAnA, the PSQI score was significantly greater among patients with AA compared to matched controls (7 ± 4.13 vs. 3.53 ± 1.96, *p* < 0.001). A greater number of AA patients depicted excess daytime sleepiness, measured with the Epworth Sleepiness Scale, than controls. Furthermore, sleep quality was worse among AA patients also suffering from anxiety or depression, thereby highlighting the importance of addressing both sleep quality and concomitant psychiatric distress in the management of AA [82].

Furthermore, a 2018 study including 25,800 with diagnosed sleep disorders and 129,000 control subjects found those with sleep disorders to have a significantly greater risk for AA than controls [83]. The authors found an adjusted hazard ratio of 1.651 among those with sleep disorders (95% CI 1.382–1.974), portraying sleep disorder as an independent risk factor of AA [83].

#### Circadian Rhythm and Clock Genes

The circadian rhythm is an internal clock of approximately 24 h that regulates alert and sleep cycles and responds to environmental changes of light [84]. The circadian rhythm is further regulated by clock genes encoding for clock proteins, which contribute to various positive and negative feedback loops. The core of the circadian clock genes lie with bHLH-PAS transcriptional activators CLOCK and BMAL1 [84]. Following heterodimer formation, CLOCK and BMAL1 activate period genes (PERs) and cryptochrome circadian regulator genes (CRYs), which translocate into the nucleus and inhibit BMAL1 and CLOCK transcriptional activity in a negative feedback loop. By inhibiting BMAL1 and CLOCK transcriptional activity, PERs and CRYs effectively inhibit their own expression, resulting in the re-activation of BMAL1/Clock. This feedback loop allows for rhythmic expression characteristic of the circadian rhythm.

Interestingly, clock genes have been found to play an important regulatory role in the hair growth cycle [85]. Furthermore, circadian clock expression changes correlate with hair growth cycle events, with the highest expression characteristic of the telogen-anagen transition. Specifically, CLOCK/BMAL1 target genes such as PEers, Dbp, and Rev-Erbα have been found to increase in telogen and early anagen [85]. In situ hybridization studies found that rhythmic circadian gene expression occurred more prominently in the secondary hair germ, which contains cycling stem and progenitor cells, in comparison to the bulge region and dermal papilla regions in which it is situated between.

Interestingly, while circadian amplitude reduced within the hair follicle proper during anagen progression, circadian amplitude remained robust in the dermis and interfollicular epidermis. Suspension of the circadian rhythm in the highly proliferative hair follicle proper corresponds to similarly observed circadian rhythm suspension in the testis and thymus, both of which are highly proliferative and differentiating tissues [85].

In 2010, Geyfman and Andersen analyzed CLOCK and BMAL1 mutant murine models and observed a significant delay in anagen progression, which was more pronounced in BMAL1 mutant mice [85]. Mutant mice entered anagen at the same time, although they experienced a week-long delay in the first anagen phase prior to resumption of the hair cycle; absence of mitotic cells in the early anagen phase was observed in mutant mice hair follicles, likely mediated by absent phosphorylated retinoblastoma protein, which fosters cell cycle progression through the G1-S checkpoint. Further analysis revealed that increased inhibitory p21 may contribute to G1-S cell cycle arrest. However, no abnormalities were observed in anagen follicle structure in mutant mice, leading authors to conclude that circadian clock genes are involved in the timing of the telogen-anagen transition, rather than hair follicle morphogenesis [85].

### 2.6. Cell Division Inhibiting Medication

Similarly, medications directly inhibiting cell division, such as various chemotherapies, can have similar effects on the cell cycle. Chemotherapy drugs, such as paclitaxel, docetaxel, vinblastine, and vincristine function to inhibit mitosis and thereby reduce the dividing capacity of rapidly growing cancer cells. However, due to lack of selectivity for cancer cells, such drugs can impact rapidly dividing cells throughout the body, including dermal papilla cells and epithelial cells of the hair follicle, in addition to matrix keratinocytes. Unsurprisingly, the highly proliferative anagen phase is most sensitive to toxins and drugs, in contrast to the mitotically inactive phases catagen and telogen. Furthermore, in addition to directly affecting cellular proliferation during the anagen phase, chemotherapy can accelerate the transition to telogen [86].

Upon termination of the drug, hair regrowth can occur, although with an occasionally different color or texture [87]. Despite reversibility, alopecia secondary to cell division inhibiting medication is an important, emotionally-distressing side effect for cancer patients; almost half of female patients consider hair loss the most traumatic aspect of chemotherapy, with fear of hair loss prompting declination of chemotherapy by 8% [88].

Unfortunately, there are no approved pharmacologic remedies for chemotherapy-induced alopecia. While topical minoxidil has been shown to reduce the severity and shorten the duration of drug-induced hair loss, it could not prevent alopecia [88]. However, scalp cooling has been shown to decrease drug delivery to the scalp, thereby mitigating chemotherapy-induced hair loss [89].

### 2.7. History-Taking Tips

As a variety of factors can promote anagen to telogen transition and contribute to hair loss, it is essential to take a thorough history for patients presenting with hair loss. Harrison and Bergfeld (2009) recommend obtaining the following information:

- Duration of hair shedding;
- Episodic or continuous patterns;
- Estimated percent hair loss;
- Potential triggers and temporal relationships;
- Recent surgery, fever, illness, childbirth, psychological stress;
- History of chronic disease, malignancy, infection, autoimmune disease, liver or renal disease;
- Menstrual history;
- Hair care products and procedures;
- Dietary history including vitamins and supplements;
- Family history of AnA, AA, autoimmune disease, or thyroid disorder;
- Medication history including botanicals;
- History of radiation therapy or heavy metal exposure.

In addition, we recommend inquiring about sleep patterns. Evaluation and lab testing utility will be discussed in Section 4.

## 3. Factors Increasing Telogen to Anagen Transition and Hair Growth

There are a variety of factors that conversely mediate telogen to anagen transition and thereby support hair growth, including increased blood flow, direct stimulation of the hair follicle, and growth factors (Table 1).

### 3.1. Increased Blood Flow

Developing hair follicles are surrounded by deep dermal vascular plexuses. Associated blood vessels function to supply nutrients to the developing follicle and foster waste elimination. As such, proper blood supply is necessary for effective hair follicle growth, further exemplified by the angiogenic properties of the anagen phase [90].

#### 3.1.1. Scalp Massage

Theoretical benefits of increased blood flow to the hair follicles justifies the assessment of scalp massage on hair parameters. A 2016 study assessed the effect of a 4-min standardized daily scalp massage for 24 weeks among nine healthy men [91]. Authors found scalp massage to increase hair thickness, upregulate 2655 genes, and downregulate 2823 genes; hair cycle-related genes including NOGGIN, BMP4, SMAD4, and IL6ST were among those upregulated, and hair-loss related IL6 was among those downregulated. The authors thereby concluded that a standardized scalp massage and subsequent dermal papilla cellular stretching can increase hair thickness, mediated by changes in gene expression in dermal papilla cells [91].

In addition, of 327 survey respondents attempting standardized scalp massages following demonstration video, 68.9% reported hair loss stabilization or regrowth [92]. Positive associations existed between self-reported hair changes and estimated daily minutes, months, and total standardized scalp massage effort. This study is limited based on recall bias and reliance on patient adherence and technique, although it suggests promising therapeutic potential for standardized scalp massage, which functions to increase blood flow.

#### 3.1.2. Minoxidil

Similarly, minoxidil, a pharmacologic agent that relaxes blood vessels and increases blood flow, has been widely utilized for the management of AnA. While topical minoxidil has been FDA approved for MPAnA and FMPAnA, oral minoxidil, especially in a low dose, is used off-label for AA and TE [93,94,95].

In addition to the relaxation of blood vessels, minoxidil also acts as an anti-inflammatory agent, an inducer of the Wnt/β-catenin signaling pathway, and as an antiandrogen [96]. Effects on anagen and telogen phases have been proposed, although a study in rats found that topical minoxidil increased DNA synthesis rate in the anagen bulb, rather than prolonging the length of the anagen phase [97]. However, animal studies have described shortened telogen and increased telogen to anagen transition [98].

A comprehensive review of oral and topical minoxidil found that 2% topical minoxidil prompts hair regrowth in both frontotemporal and vertex areas among males with MPAnA, with peak hair regrowth after one year of use [96]. No significant differences were found between 2% and 5% topical solutions in terms of efficacy. A meta-analysis assessing topical minoxidil found an average score difference of 16.7 for the promotion of total hair growth between individuals receiving topical minoxidil vs. control (95% CI 9.34–24.03). An average difference of 20.9 (95% CI 9.07–32.74) was observed for non-vellus hair growth [99]. Similarly, individuals using minoxidil had a 2.28× greater likelihood of exhibiting hair growth than those using a placebo (95% CI 1.343–1.80).

In addition, despite off-label use, oral minoxidil 5 mg/day exhibited significantly greater efficacy than both 2% and 5% topical minoxidil in males with MPAnA [96]. Low dose oral minoxidil and sublingual may additionally be safe and effective in patients with FPAnA [96]. Interestingly, a review of 17 studies with 634 patients found oral minoxidil to be an effective strategy among patients refractory to topical formulations [100].

Despite minoxidil efficacy, authors have sought therapeutic strategies to maintain biological efficacy while reducing side effects, such as hypertrichosis. For example, a 2022 retroactive study of patients with minoxidil-induced hypertrichosis found clear improvement among 35 FPAnA patients following initiation or up-titration of oral bicalutamide, an antiandrogenic medication [101]. Simultaneous bicalutamide treatment at a mean dose of 14.4 mg allowed an increase in the mean daily minoxidil dose without the development of hypertrichosis.

In addition, authors have sought novel minoxidil delivery methods to maximize effects while minimizing side effects. A 2022 study used biocompatible and safe hyaluronic acid (HA)-constructed microneedles to deliver minoxidil to hair dermal papilla cells [102]. A chemotherapy-induced alopecia murine model was used to examine the effects of HA-microneedle delivery of minoxidil compared to conventionally applied minoxidil. HA solution alone demonstrated reduced hair loss in mice with alopecia. Yet, authors observed maximal anti-alopecia effects with minoxidil loaded HA-microneedles, measured via hair follicle length, hair density, and dermal thickness, although efficacy was comparable with topical minoxidil treatment [102]. Despite similar efficacy, microneedle delivery of minoxidil may maximize anti-alopecia effects while minimizing side effects during treatment.

Lastly, a 2022 study assessed the efficacy of liquid crystal nanocarriers to direct minoxidil to the pilosebaceous follicle, which is difficult to reach given its origination in deeper skin layers [103]. Authors loaded minoxidil into the liquid crystal nanocarrier and assessed biological effectiveness compared to conventionally applied minoxidil among rats. The crystal nanocarrier selectively targeted the pilosebaceous follicle, increasing efficacy and duration of biological effects while reducing side effects. Whereas untreated rats depicted a mean 3.6 mm regrowth and rats treated with hydro-alcoholic 5% *w*/*v* minoxidil showed a mean 4.3 mm regrowth after one month, rats treated with minoxidil-loaded nanocarriers demonstrated a significantly (*p* < 0.001) greater mean re-growth (5.6 mm). The percentage of hair length increase was 19% and 59% for rats treated with hydro-alcoholic minoxidil and minoxidil-loaded nanocarriers, respectively. In addition, 12 healthy human volunteers demonstrated tolerability and safety of the nanocarrier via a safety evaluation characterized by treatment application on five ventral surfaces of each forearm [103]. This study suggests the liquid crystal nanocarrier is a safe and effective vehicle to delivery minoxidil selectively to the pilosebaceous follicle, allowing reduced concentrations of active compound to achieve greater biologic efficacy.

#### 3.1.3. HIF-1α

Hypoxia inducible factor (HIF) is a transcription factor that responds to hypoxic stress via angiogenesis regulation. As dermal papilla cells are reactive to hypoxia, HIF stimulation modulates neovascularization and regeneration, which is necessary to combat the lack of blood vessel and nutrient supply characteristic of AnA [104]. Thus, a 2023 study assessed the effect of HIF strengthening factor (HSF) hair restoration on various hair parameters [104]. Twenty subjects, four female and sixteen male, underwent a once-daily application of HSF hair restoration technology for nine months. Authors observed a 7.2% increase in hair thickness, 14.3% increase in hair density, and a 20.3% increase in shine and elasticity. Treatment-responsive subjects (85% of the cohort) depicted a 66.8% reduction in hair loss after six months of treatment, with an increase in hair growth up to 32.5% (mean 1.8%). Lastly, the test area depicted an average anagen hair percent increase of 8.0% and an average telogen hair percent decrease of −14.0%, depicting the ability of HSF hair restoration technology to promote telogen to anagen transition [104].

### 3.2. Direct Stimulation of the Hair Follicle

Herbs, supplements, prostaglandins, and light-based approaches have been shown to promote hair growth via direct stimulation of the hair follicle.

#### 3.2.1. Herbs and Phytochemicals

A review article conducted in 2019 summarized a variety of clinical trials that assessed the use of herbs for the treatment of hair loss [105]. The most evidence for promoting hair growth was attributed to many herbs including, “*Curcuma aeruginosa* (pink and blue ginger), *Serenoa repens* (palmetto), Cucurbita pepo (pumpkin), *Trifolium pratense* (red clover), and *Panax ginseng* (Chinese red ginseng)” [105]. The article states that the beneficial effects on hair growth from these herbs is possibly due to their inhibitory effects on 5-alpha-reductase.

An additional review study, also conducted in 2019, summarized different alternative remedies for the treatment of alopecia [106]. Among the herbal treatments described, it was noted that *Curcumin aeruginosa*, when used in combination with minoxidil, can provide synergistic hair growth effects. Multiple studies summarized also supported the efficacy of topical melatonin, with results indicating that melatonin can increase hair counts, hair density, and anagen hair. Five studies also consistently supported the use of capsaicin for hair growth. One study described increased hair growth with oral supplementation and the remaining studies utilized topical capsaicin, which also displayed increases in hair growth.

Furthermore, *Morbus alba*, otherwise known as white mulberry, is an herb that has been shown to influence the hair growth cycle [107]. A study conducted in 2021 on hair follicle dermal papilla cells (HFDPCs) displayed promising results. *Morbus alba* was found to cause activation of beta-catenin in HFDPCs which subsequently caused activation of the anagen phase. This finding supports the potential use of *Morbus alba* as a possible treatment option for hair loss.

Bhrinjaraj, otherwise known as *Eclipta alba*, has also shown promising effects on hair growth. A study was conducted on male albino rats, and they received either topical Eclipta alba formulated into a 5% petroleum ether extract or the positive control, Minoxidil 2% [108]. The results showed that the treatment group with Eclipta alba had higher counts of hair follicles in the anagen phase compared to the control.

Additionally, quercetin, which is a component of *Hottuyunia cordata* extract, has also shown to have beneficial effects for hair growth. A study conducted in 2020 utilized human dermal papilla cells (hDPCs) to test the effects of the extract [109]. They found significant effects on the function of mitochondria. Specifically, the mitochondrial membrane potentials and NADPH production was found to be increased, suggesting enhanced mitochondrial function. Furthermore, Bcl2 expression increased which is a marker for the anagen phase and increases cell survival. The expressions of the following were also found to be increased: Ki67 (cell proliferation marker), various growth factors such as VEGF, bFGF, KGF, and phosphorylation of Akt, Erk, and CREB. The extract was found to increase hair shaft growth, specifically in cultured human hair follicles. Overall, the researchers attributed the increased hair growth to the activation of the MAPK/CREB pathway which led to the increased expression of growth factors due to quercetin application.

Another study testing quercetin in mouse models further supported the beneficial effects on hair growth [110]. Mice with alopecia areata were given either quercetin or placebo injections. The results showed that the mice receiving the quercetin injections had improved hair growth in lesioned areas whereas the placebo group did not. The researchers also utilized non-alopecic mice and heat-treated them to induce alopecia; placebo or quercetin injections were then provided. They found that none of the mice receiving quercetin injections developed alopecia, whereas 24% of the placebo group did develop alopecia. Thus, quercetin may be a viable treatment option for treating alopecia although additional studies in humans are warranted.

Rosemary oil is another herbal remedy that has been suggested to increase hair growth. A study conducted in 2015 recruited 60 patients and assigned them to either use topical minoxidil 2% or rosemary oil for 6 months. By the end of the study both groups displayed significant increases in hair counts (*p* < 0.05) compared to baseline, although there was no significant difference between the two groups. Nevertheless, rosemary oil in this study showed comparable results to minoxidil. Interestingly, minoxidil also was observed to be more commonly associated with scalp itching (*p* < 0.05) [111]. Lavender oil (LO) has also been tested as a hair growth remedy. A study conducted in 2016 with mouse models assessed 3% LO vs. 5% LO vs. 3% minoxidil applied topically on the backs of mice once a day, 5 days per week for 1 month. They found that hair follicles significantly increased in all 3 groups by the end of the study, however, they did not comment on the difference among the groups [112].

Proanthocyanins have also shown promising results for hair growth in the literature. A study conducted on mouse hair follicle cells found that proanthocyanins extracted from grape seeds caused a 230% increase in proliferation compared to the control vehicle. The authors attribute the hair growth effects to the proanthocyanins ability to increase transition from the telogen phase to the anagen phase [113]. Another study studied the effects of procyanidin B2 derived from apple extract. Thirty male subjects with male-pattern hair loss were recruited and instructed to apply either 1% procyanidin B-2 or placebo to the scalp twice daily for 6 months. Hair density at the end of the study was significantly higher in the treatment group (*p* < 0.0001) [114].

Overall, there are many herbs that have been tested in the literature for their effectiveness in treating alopecia. Many of these trials have found promising results, and thus they provide another treatment modality for patients experiencing hair loss to utilize.

#### 3.2.2. Supplements

Supplements for hair growth have also been heavily researched for hair growth. In a randomized controlled trial conducted in 2018, 40 women with self-perceived hair thinning were recruited to either take the herbal supplement (brand: Nutrafol) or placebo for 6 months [115]. The supplement was noted to include a variety of ingredients including curcumin, ashwagandha, and saw palmetto. By days 90 and 180, the treatment group experienced a significant increase in the terminal and vellus hair counts compared to the placebo (*p* < 0.009). Another supplement composed largely of marine protein (brand: Viviscal) were also tested in a separate randomized placebo-controlled trial [47]. Participants included 60 women with thinning hair and were asked to take either placebo or the supplement twice daily for 3 months. The results showed a significant increase in the terminal hair counts in the treatment group compared to placebo (*p* < 0.0001).

Pumpkin seed oil supplements have also been shown to be beneficial for hair loss. A randomized control trial including 76 males with androgenetic alopecia were instructed to either take 450 mg of pumpkin seed oil supplements or placebo for 24 weeks [116]. Hair counts improved by 40% in those taking pumpkin seed oil whereas hair counts only improved 10% in the placebo group (*p* < 0.001). The exact mechanism in the hair cycle is not known, however it is thought that pumpkin seed oil is enriched for delta-7-phytosterols and may inhibit 5-alpha-reductase activity [117].

#### 3.2.3. Light-Based Approaches

Low level light therapy refers to therapeutic exposure to low levels of red and near infrared light [118]. Studies have demonstrated increased hair growth in mice with chemotherapy-induced alopecia and AA, in addition to both men and women human subjects. Proposed mechanisms of efficacy include stimulation of epidermal stem cells residing in the hair follicle bulge and promoting increased telogen to anagen phase transition [119]. Interestingly, while minoxidil and finasteride are the only FDA-approved drugs for AnA, a 2017 study found comparable efficacy among patients receiving low-level light therapy versus topical minoxidil among patients with FPAnA [120]. In addition, combination therapy resulted in the greatest patient satisfaction and lowest Ludwig classification scores of AnA.

A meta-analysis including eleven double-blinded randomized controlled trials found a significant increase in hair density among patients with AnA receiving low level light therapy compared to those in the placebo-controlled group; the standardized mean difference (SMD) was 1.316 (95% CI 0.993–1.639) [121]. Low level light therapy was effective for men and women. Furthermore, a subgroup analysis observed a more significant increase in hair growth in those receiving low-frequency therapy (SMD 1.555, 95% CI 1.132–1.978) than receiving high-frequency therapy (SMD 0.949, 95% 0.644–1.253) [121]. Despite the limitation of the heterogeneity of included trials, these results suggest low level light therapy to be a promising therapeutic strategy for AnA [121], although further research is necessary to determine the optimal wavelength and dosimetric parameters for hair growth [119].

#### 3.2.4. Prostaglandins

Latanoprost is a prostaglandin F2 agonist and has been shown to have a direct effect on hair growth and pigmentation in eyelashes and hair around the eyes [122]. Clinically used to treat glaucoma, this medication was found to affect the follicles in the telogen phase and cause them to move to the anagen phase; this was supported by the increased number and length of eyelashes seen in patients using latanoprost [122]. Subsequently, the application of latanoprost for patients experiencing alopecia was assessed in clinical studies. One conducted in 2012 studied the effects of 0.1% latanoprost solution applied to the scalp for 24 weeks [123]. Participants included 16 males with mild androgenetic alopecia and were instructed to apply placebo on one area of the scalp and the treatment on another area. The results indicated that the area of scalp receiving latanoprost had significantly improved hair density compared to placebo (*p* < 0.001).

Another prostaglandin known as bimatoprost, a prostamide-F2 analog, was also found to have a positive effect on hair growth in human and mouse models. A study conducted in 2013 also found that bimatoprost, in both humans and mice, stimulated the anagen phase of hair follicles prompting an increase in hair length, i.e., promoting hair growth [124]. The study also confirmed the presence of prostanoid receptors in human scalp hair follicles in vivo, opening the strong possibility that scalp follicles can also respond to bimatoprost in a similar fashion.

It is important to note, however, that not all prostaglandins induce hair growth. In a study analyzing individuals with androgenetic alopecia with a bald scalp versus a haired scalp, it was discovered that there was an elevated level of prostaglandin D2 synthase at the mRNA and protein levels in bald individuals [125]. They were also found to have an elevated level of prostaglandin D2. When analyzing the level of prostaglandin D2 synthase presence through the various phases of hair follicular growth, it was found that the level steadily increased throughout the anagen phase with a peak in late anagen, at the time of transition to the catagen (breakdown) phase. Therefore, the study concluded that PGD2’s hair loss effect represents a counterbalance to PGE2 and PGF2’s hair growth effects. In conclusion, prostaglandins are a promising treatment option for alopecia that require larger clinical studies; however, clinicians should be aware of which one to recommend for hair growth, as not all prostaglandins are alike.

### 3.3. Growth Factors and Platelet Rich Plasma

Platelet rich plasma (PRP) has conventionally been used to supplement a patient’s endogenous platelet supply to promote increased healing. However, its prominent supply of growth factors has prompted assessment of PRP for alopecia. Growth factors promote hair growth and increase the telogen to anagen transition. For example, a murine study found the fibroblast growth factor (FGF) induced the anagen phase and subsequently promoted hair growth [126]. Growth factors prominently included in PRP include platelet-derived growth factor (PDGF), transforming growth factor β (TGF-β), vascular endothelial growth factor (VEGF), epidermal growth factor (EGF), insulin-like growth factor (IGF) and FGF [127].

The growth factors of platelet-rich plasma stimulate the development of new follicles and neovascularization [128]. Three meta-analyses have assessed the efficacy of PRP injections compared to placebo control on the number of hairs per cm^2^ among patients with AnA. One meta-analysis involving 177 patients found a mean improvement of PRP treatment compared to placebo of 17.9 (95% CI 5.8–30.5, *p* = 0.004) [129]; a second meta-analysis with 262 AnA patients observed a mean difference of 38.8 (95% CI 22.22–55.28, *p* < 0.00001) [130]; and a third meta-analysis including studies with parallel or half-head design found a mean difference of 30.4 (95% CI 1.77–58.93, *p* < 0.00001) [131].

Despite the efficacious results described by each meta-analysis for the use in AnA, gender differences have been observed. A 2020 meta-analysis found that while PRP significantly increased hair density and hair diameter from baseline in men, PRP only increased hair diameter in women, in the absence of significantly increased hair density. Furthermore, hair density in men was only significantly increased by a double spin method, in contrast to a single spin method [132]. The authors conclude that PRP effectiveness may be improved via higher platelet concentrations. Ultimately, PRP injections appear to have clinical efficacy in early studies albeit slightly different effects in men vs. women. Future research is necessary to establish the optimal treatment protocol for both men and women with AnA. Also, the role of diet in the days prior to collection of the PRP has not been assessed in conjunction with hair, although diet influences the quality of the PRP [133].

## 4. Diagnostic Lab Testing

### 4.1. Ferritin

Iron is a mineral that is integral for the body. It allows for humans to produce hemoglobin and myoglobin which are essential for the distribution of oxygen within the body. Additionally, iron plays a role in the production of certain hormones and allows for normal growth and development. Ferritin is a protein that allows for the intracellular storage of iron as, without it, iron intracellularly can produce free radicals which can damage cell machinery. Serum ferritin levels can be a marker for overall iron storage levels in the body [134]. Low serum ferritin levels have been supportive of an iron deficiency, anemia most commonly, however low levels can also be found in hypothyroidism and ascorbate deficiency [134].

Clinically, studies have suggested the correlation of low ferritin levels with hair loss. Although the mechanism of how low ferritin may lead to hair loss is not known, one theory highlights the importance of iron as a cofactor for ribonucleotide reductase, which is the rate limiting enzyme in DNA synthesis [135]. Since hair follicle cells are rapidly dividing, they require the constant use of ribonucleotide reductase and a deficiency of iron may limit the efficiency of this enzyme. In turn, this can lead to decreased cell turnover and regeneration leading to decreased hair growth. Thus, the evaluation in a patient presenting with hair loss has often involved an assessment of iron levels [135].

#### 4.1.1. Premenopausal vs. Postmenopausal Women

Several studies have investigated the relationship of low ferritin levels and hair loss. One study performed by Rasheed et al. evaluated 80 premenopausal women [66]. Females aged 18–45 years were included in the study. The serum ferritin levels were assessed in 80 women who had telogen effluvium (TE) or female-pattern hair loss (FPHL), and in 40 women with no hair loss. The average ferritin levels in women with TE was 14.7 μg/L and 23.9 μg/L in those with FPHL; the control group had average ferritin levels of 43.5 μg/L. The average ferritin levels in both types of hair loss were significantly lower when compared to controls (*p* < 0.001). Another study conducted in 2022 explored ferritin levels in premenopausal and postmenopausal women with FPHL [136]. Statistically significant lower ferritin levels <70 μg/L were found only in premenopausal women with FPHL (*p* = 0.01).

Furthermore, another study conducted in 2013 also found significantly low levels of ferritin only in premenopausal women with FPHL [137]. The average serum ferritin level in premenopausal women was 30.67 μg/L and this was compared to age/sex matched healthy controls who had an average ferritin level of 69.32 μg/L (*p* < 0.001). Postmenopausal women, on the other hand, had an average ferritin level of 83.22 μg/L and when compared to their age/sex matched healthy controls who had an average ferritin of 85.38 μg/L, there was no statistically significant difference. Thus, overall, many studies seem to consistently highlight a more significantly lowered ferritin level in premenopausal women with FPHL. This may be explained by the fact that iron deficiency tends to be more common in premenopausal women due to monthly blood loss attributed to menstruation [138]. Although much less common, iron deficiencies can also occur in postmenopausal women due to malabsorption or gastrointestinal bleeding; however, there may be other factors contributing to their hair loss which can explain the lack of statistically significant changes in the ferritin level [135].

An important fact to highlight, however, is that it is difficult to conclude whether or not a low serum ferritin level is correlated to hair loss in postmenopausal women as most of the studies have been performed only with premenopausal women. Further investigation is required specifically in postmenopausal women with large sample sizes to better understand the role of ferritin in their hair loss.

#### 4.1.2. Men vs. Women

Because the literature has widely highlighted the importance of ferritin levels in regards to hair loss, a few studies have been performed to determine if low ferritin levels are also significant in males experiencing hair loss. In the study described previously by Tahlawy et al., the researchers also assessed 30 males with androgenetic alopecia and compared their serum ferritin levels with 30 healthy males [66]. The results showed no statistically significant differences in ferritin levels in patients with androgenetic alopecia compared to controls. Furthermore, the study described previously by Park et al. also assessed ferritin levels in 97 males with male-pattern hair loss (MPHL). The average ferritin levels in males with MPHL was 132.3 μg/L which was significantly lower than the average found in controls, 210.2 μg/L (*p* < 0.001); however, it is important to note that both of these levels are still considered to be in the normal serum ferritin range. As described previously, the women in this study did show an abnormally low average serum ferritin level in those with FPHL.

In general, based on the current studies it is challenging to make any conclusions regarding the involvement of ferritin in hair loss experienced by males. There are very few studies overall which have assessed ferritin levels in males with alopecia and, in the ones currently described, there seems to be no major significant correlation of ferritin levels to alopecia, especially when compared to the strong correlations found in women. Thus, further investigation is warranted to determine the importance of ferritin in males before clinicians can make any treatment recommendations.

### 4.2. ANA

Antinuclear antibody (ANA) is a common lab marker that tests for the presence of an antibody against material within the nucleus of the cell. Its most clinical value has been in the diagnosis of systemic lupus erythematosus; however, the marker has been found to be commonly positive in numerous other autoimmune diseases including polymyositis, dermatomyositis, Sjogren’s syndrome, rheumatoid arthritis, scleroderma, and mixed connective tissue disease. As a result, obtaining an ANA level is more often used as a supplement to making a diagnosis; the clinical signs and symptoms play a more integral role to correctly diagnosing which disease a patient may have since an ANA positive test could occur in a variety of diseases [139]. Importantly, a positive ANA is estimated to be prevalent in 25% of the population, including healthy individuals. Many studies have shown ANA positivity in individuals with no signs or symptoms of rheumatologic disease. Therefore, its utility has been extremely controversial.

The utility of obtaining ANA markers for patients presenting with hair loss is unclear. A retrospective study was conducted in 2015, with 49 women and 56 men presenting with pattern hair loss [140]. The researchers found the ANA to be positive in 19.1% of the women and 11.3% of the men, with a total of 30.4% ANA positivity. Thus, the ANA was found to be significantly more positive in women (*p* < 0.05). When comparing the severity of hair loss using the BASP classification, there were no statistically significant differences among those with a positive ANA and those with a negative one. Additionally, there was no significant difference in average hair density or hair shaft diameter between ANA positive and negative patients. Thus, although many patients were found to incidentally have a positive ANA, it is unclear whether that has any correlation to their hair loss.

In general, obtaining ANA lab markers should currently be limited only to those patients with a high clinical suspicion of having a rheumatologic or autoimmune disease [141]. Additional studies must be performed with larger sample sizes of various types of alopecia to obtain a better understanding of its role and importance. Based on the current literature, since ANA positivity seems to be relatively prevalent in the population, a positive test in an otherwise asymptomatic person may have low clinical utility [139,142].

### 4.3. RPR

Rapid plasma reagin (RPR) is a test that can be utilized to diagnose syphilis, a sexually transmitted infection caused by *Treponema pallidum* bacteria [143]. There are many stages during the infection that each present with specific symptoms. These include primary-, secondary-, and tertiary-stage syphilis. Of importance to hair loss is the secondary stage. Syphilitic alopecia (SA) is defined by the occurrence of diffuse or patchy hair loss and often has been described as having a “moth-eaten” appearance [144]. Interestingly, SA can mimic various other forms of alopecia including telogen effluvium and alopecia areata [145]. As a result, it may be easy to miss a diagnosis of syphilis if the patient has not experienced other typical symptoms of syphilis. The literature has described cases where the only clinical manifestation has been hair loss [145]. As a result, it will be important for clinicians to also consider a sexual history from patients presenting with hair loss and include RPR testing in the work-up if that seems appropriate.

### 4.4. Thyroid Hormones

One of the known presenting symptoms of hypothyroid and hyperthyroidism is hair loss. There are thyroid hormone receptors present in human skin cells, therefore any alterations in the quantity of thyroxine or triiodothyronine will lead to an alteration in human skin and hair follicles [28]. In a study analyzing how T3 and T4 directly influence human hair follicles in vitro, it was found that both T3 and T4 have an inhibitory effect on the apoptosis of human hair matrix keratinocyte cells, while T4 was also found to have a significant stimulatory effect on their proliferation [146]. T3 was not found to have a significant stimulatory effect on the keratinocytes. Furthermore, the study found that increased levels of thyroid hormones had a direct correlation with increased numbers of anagenic hair follicles, and a decrease in catagenic hair follicles. Finally, T3 and T4 were also both found to have a stimulatory effect on hair follicle pigmentation. Overall, the study concluded that both T3 and T4 alter key parameters in human hair follicle growth and support the claim that the deficiency of thyroid hormones in hypothyroid individuals directly plays a role in the symptomatic hair loss.

### 4.5. Functional Testing

#### 4.5.1. Diurnal Cortisol Slope Testing

Diurnal cortisol slope testing is a functional lab test that assesses the change in cortisol levels throughout one day. Cortisol is the main glucocorticoid hormone released in response to both acute and chronic stress. It has numerous effects on the body including immune function suppression, activation of the sympathetic nervous system, and alter glucose homeostasis [147]. Although acutely these effects allow the body to adequately function, chronically these changes can be detrimental and lead to inflammation, fatigue, and psychological maladaptation [148].

Cortisol levels can be assessed through saliva sample collections that a patient collects through the course of one day. A normal diurnal cortisol rhythm follows a distinct pattern throughout the day. As outlined by Adam et al., the first sample taken is to assess the waking cortisol, which is defined as the level established immediately upon awakening in the morning; this level is normally high [149]. The next sample is taken 30–40 min after waking up and is called the cortisol awakening response; this level will normally display a surge compared to the waking cortisol. The remainder of samples are collected at varying time points in the day, but overall should display a decline with the lowest levels recorded near bedtime. Overall, these cortisol levels can be used to generate a diurnal cortisol slope. Any changes or flattening in the curve of the slope can indicate abnormal cortisol production. Studies have shown that abnormal cortisol rhythms throughout the day can be associated with numerous negative health outcomes and an imbalance of the hypothalamic pituitary adrenal axis (HPA) [149]. However, this has not been studied specifically in relation to hair loss or hair thinning. Thus, diurnal cortisol slope testing may be beneficial to determine if abnormal cortisol rhythms are contributing to hair loss, as part of future studies.

##### Correlations to Hair Cortisol

A novel method to assess the function of the HPA axis and cortisol levels is to obtain hair cortisol levels. This method requires obtaining a strand of hair which is then ground or minced to extract cortisol levels [150]. Interestingly, this method to assess cortisol levels provides a few key differences from the traditional diurnal cortisol slope testing. First, hair cortisol levels do not provide an acute snapshot of cortisol activity much like the traditional diurnal cortisol slope testing provides, but rather it offers a retrospective look into the history of what cortisol levels have been like in the body. On average, since hair grows at a rate of 1 cm/month, the literature has outlined that the most proximal 1 cm of a hair strand to the scalp provides information about the cortisol activity in the last month [151]. The second centimeter of hair provides information about 2 months prior and the next centimeter provides details about 3 months prior and so on. The hair cortisol levels are considered reliable up to 6 cm from the scalp. Additionally, since this method only requires the extraction of hair strands, it could be more reliable than traditional cortisol testing which is highly dependent on patient compliance to be accurate [151].

One major drawback of hair cortisol testing and its correlation to hair loss is that there have not been many studies that have included it in their methodology for assessing hair loss, specifically. The current literature is limited to only highlighting, thus far, that hair cortisol testing is a reliable biomarker indicating that the body is undergoing chronic stress [152]. However, no conclusions can be made from that information regarding its utility in hair loss.

Current studies have focused on testing hair cortisol in rhesus macaques, a species of monkey, experiencing alopecia. A study conducted in 2014 with 99 rhesus macaques monkey’s divided them into two groups [153]. The alopecia group included monkeys with 30% or more hair loss and the control group included monkeys with less than 5% hair loss. Hair cortisol levels were analyzed in both groups and results showed that the alopecia group had increased concentrations of hair cortisol compared to the control group. Although this study provides a foundation for the incorporation of hair cortisol as a tool for understanding hair loss, further research is still warranted, especially in humans. Overall, it is too early to determine if hair cortisol testing may be beneficial in the work-up for patients presenting with hair loss.

#### 4.5.2. Mitochondrial Function Testing

##### Thyroid Impact on Mitochondrial Function

Thyroid hormones are major regulators of energy expenditure within the body and are responsible for establishing a basal metabolic rate. Mitochondria are the primary organelles in cells that are involved in energy production. Thus, thyroid hormones have been widely supported by the literature as having a role in regulating mitochondrial function [154]. Many studies have suggested that thyroid hormones alter the levels of mitochondrial oxygen consumption and subsequently ATP production. Specifically, in studies investigating the effects of hyperthyroidism on mitochondria, it was collectively found that mitochondrial oxygen consumption rates were increased along with ATP production rates [155].

Interestingly, a study conducted in 2014 investigated the impact of thyroid hormones on mitochondria present in hair follicles [154]. The study utilized organ cultured human scalp hair follicles and subjected them to TSH, T3, and T4 hormones. All of the thyroid hormones were found to increase gene and protein expression of “mitochondrial-encoded subunit 1 of cytochrome c oxidase (MTCO1), a subunit of respiratory chain complex IV, mitochondrial transcription factor A (TFAM), and Porin”. Additionally, complex 1, complex 4, and mitochondrial biogenesis were each upregulated. Furthermore, the study also found that T3 and T4 hormones both decreased reactive oxygen species (ROS) production. This finding is clinically important as high levels of ROS production have been correlated to contributing to a variety of dermatologic conditions. Thus overall, this study highlights the impact of thyroid hormones on mitochondrial energetic dynamics in hair follicles. Clinically, this is important because an imbalance in the hormones could contribute to hair loss. Thus, evaluating thyroid hormones in individuals presenting with hair loss may be useful.

##### Organic Acid Testing of Krebs Cycle and Electron Transport Chain

Several studies have highlighted the effects of mitochondrial dysfunction on hair [156,157]. The mitochondria is the site of action for major biochemical reactions, including the electron transport chain and the Krebs cycle. In a study performed by Singh et al., mice with depleted mtDNA were found to have profound hair loss suggesting the importance of mitochondrial integrity [156]. Additionally, a study performed with epidermal specific Crif1 knockout mice found that the hair cycle was significantly reduced [158]. Crif1 is a mitochondrial protein responsible for the placement of oxidative phosphorylation (OXPHOS) polypeptides in the inner membrane of mitochondria. Furthermore, as discussed previously, the trial conducted by Kim et al. showed that quercetin improved mitochondria function which led to improved hair growth in cultured hair follicles [109]. Specifically, they noted increased cell proliferation markers, growth factors, increased MAPK/CREB signaling, increased NADPH production and increased mitochondrial membrane potentials, which collectively contributed to the improved hair growth noted in cultured hair follicles after supplementation with quercitrin. Thus, this study further supports the integral role of healthy mitochondrial function in the maintenance of normal hair growth.

As a result, assessing mitochondrial function may be clinically useful for determining its level of contribution to hair loss a patient may be experiencing. One method for testing the function of mitochondria is via organic acid profile testing (OAPT). The OAPT utilizes urine samples from patients to evaluate many different metabolites that can be indicators for how well the Krebs Cycle and electron transport chain (ETC) are functioning, since each of these reactions produce various byproducts [159]. Although organic acid testing is widely available in the functional medicine space, there are a dearth of studies that correlate it to hair loss or hair thinning. Therefore, given its widespread use within functional medicine, a formal clinical study on the utility of organic acid testing and hair loss should be conducted.

## 5. Conclusions

Hair growth is mediated by a complex cycle consisting of anagen, catagen, telogen, and exogen. A variety of factors impact the hair cycle, inducing anagen to telogen transition or vice vera. Inflammation has been shown to foster anagen to telogen transition and mediate a variety of hair loss subtypes via proinflammatory substance P regulation. Thyroid hormones and dihydrotestosterone exhibit regulation of the hair cycle, and research has suggested the ratio of estrogen to testosterone may be more clinically relevant than the serum levels of either hormone in isolation. While vitamin and mineral deficiency has been associated with sparse hair and alopecia, there is limited evidence to suggest supplementation in healthy subjects is beneficial. Poor sleep and cell division inhibiting medications, including various chemotherapies, negatively impact the hair cycle and contribute to loss. Conversely, increased blood flow, direct stimulation of the hair follicle, and growth factors promote telogen to anagen transition and hair growth. Specific therapies can include scalp massage, minoxidil, herbs, supplements, low level light therapy, prostaglandins, and platelet-rich plasma. Evidence is promising for the therapeutic success of many such modalities, although limitations commonly include poor study design with small sample sizes and inconsistent therapeutic protocols. A variety of diagnostic tests can be employed to determine contributing factors of hair loss. Useful testing includes serum ferritin and thyroid hormone panels. Diurnal cortisol slope testing may assess the balance of the HPA axis and the influence of stress while OAPT testing may help assess mitochondrial function in a healthy patient. ANA lab markers should only be ordered if there is suspicion for ongoing autoimmunity. There is inadequate evidence to currently suggest utility of obtaining hair cortisol levels. Ultimately, the numerous factors impacting the hair cycle necessitate a holistic and individualized approach.

## Figures and Tables

**Table 1:** Summary of interventions indicated for hair growth.

Intervention	Support of Hair Cycle	Topical Dose or Dose Range	Evidence (Humans, Animals)
Finasteride	Inhibit 5-alpha-reductase	1 mg daily	Humans (Men)–FDA Approved
Pumpkin seed oil	Inhibit 5-alpha-reductase	400 mg oral	Humans
Herbal based supplement (Nutrafol)	Anti-inflammatory, reduce Stress, and DHT inhibiting properties		Humans
Minoxidil (Topical)	Increase local blood flow	Women—3% or 5% daily Men—5% twice daily	Humans—FDA Approved
*Morbus alba*	Activation of anagen phase		Humans
Low level light therapies	Increased telogen to anagen phase transition		Humans
Latanoprost	Activation of anagen phase	0.1% latanoprost solution topical	Humans
Bimatoprost	Stimulate anagen phase	0.03% bimatoprost solution topical	Humans
Marine protein-based supplement (Viviscal)	Prolongs anagen phase	450 mg oral	Humans
Platelet rich plasma injections	Induces anagen phase		Humans
Bhringaraj (*Eclipta alba*)	Activation of anagen phase	5% petroleum ether extract topical	Mice
Quercetin	Supports mitochondrial function and anagen phase		Mice

## Abbreviations

AA:	Alopecia areata
AnA:	Androgenetic alopecia
CI:	Confidence interval
CRY:	Circadian regulator gene
DHEAS:	Dehydroepiandrosterone sulfate
DHT:	Dihydrotestosterone
FGF:	Fibroblast growth factor
FPAnA:	Female-pattern androgenetic alopecia
FSH:	Follicle-stimulating hormone
Ig:	Immunoglobulin
LH:	Luteinizing hormone
LO:	Lavender oil
MPAnA:	Male-pattern androgenetic alopecia
OR:	Odds ratio
PRP:	Platelet rich plasma
PSQI:	Pittsburgh Sleep Quality Index
SHBG:	Sex hormone binding globulin
SMD:	Standardized mean difference
TE:	Telogen Effluvium
TSH:	Thyroid-stimulating hormone
VDR:	Vitamin D receptor
ROS:	Reactive oxygen species
ETC:	Electron transport chain
OAT:	Organic Acid Testing
HA:	Hyaluronic acid
HIF:	Hypoxia inducible factor
HSF:	HIF strengthening factor
HPA:	Hypothalamic pituitary adrenal axis
SA:	Syphilitic alopecia
PER:	Period gene
RPR:	Rapid plasma reagin
ANA:	Antinuclear antibody
MPHL:	Male-pattern hair loss
FPHL:	Female-pattern hair loss
IGF:	Insulin-like growth factor
